# Supplementary figures and images for: Nutritional combinatorial impact on the gut microbiota and plasma short-chain fatty acids levels in the prevention of mammary cancer in Her2/neu estrogen receptor-negative transgenic mice
Source: PLoS One. 2020 Dec 31;15(12):e0234893. doi: 10.1371/journal.pone.0234893 (PMC7774855; doi:10.1371/journal.pone.0234893)

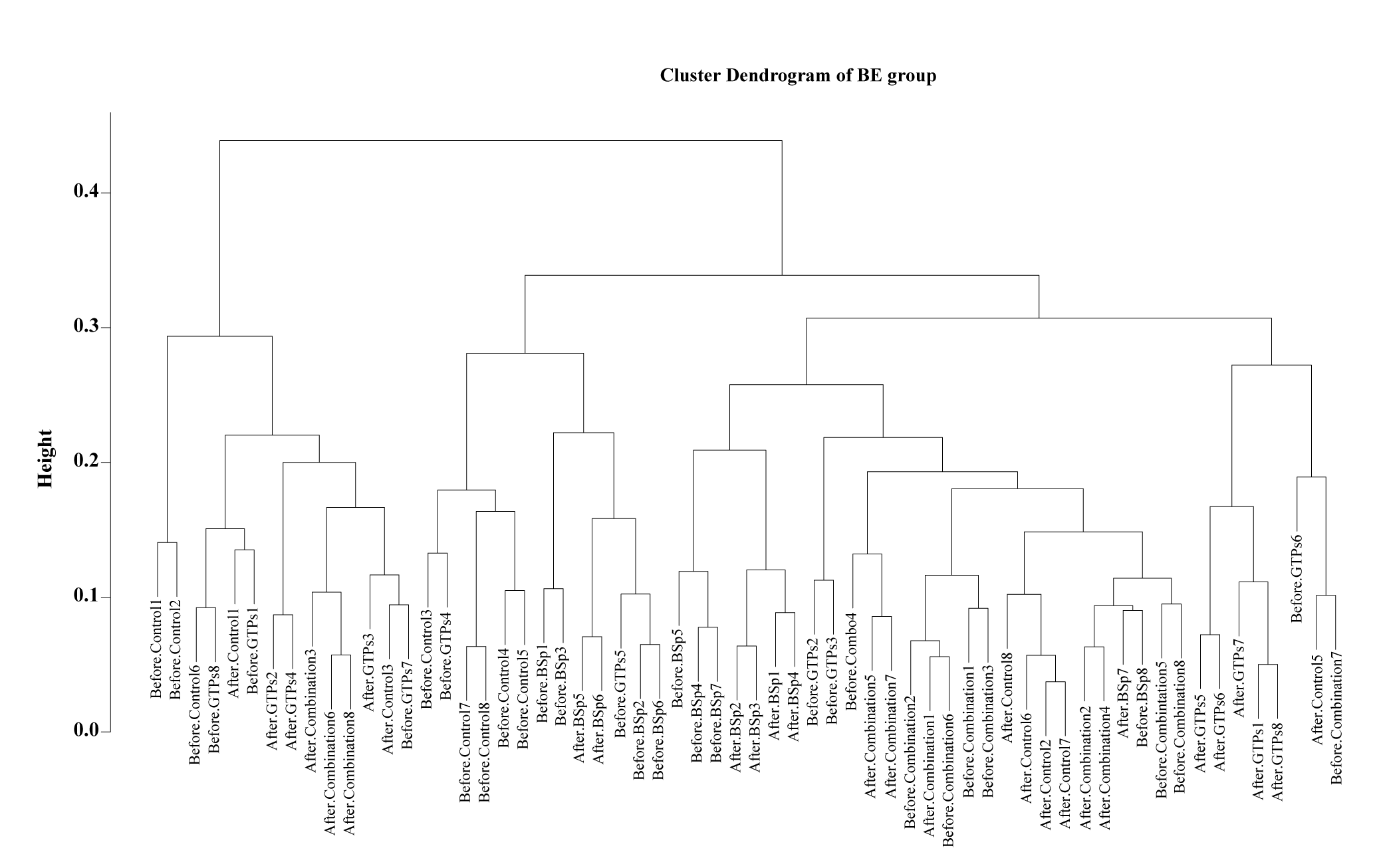

Supplement: S1 Fig — This clustering dendrogram shows no outliers and therefore all samples from dietary treatments were used in the fecal samples analyses of BE group. (TIF) [file pone.0234893.s001.tif]

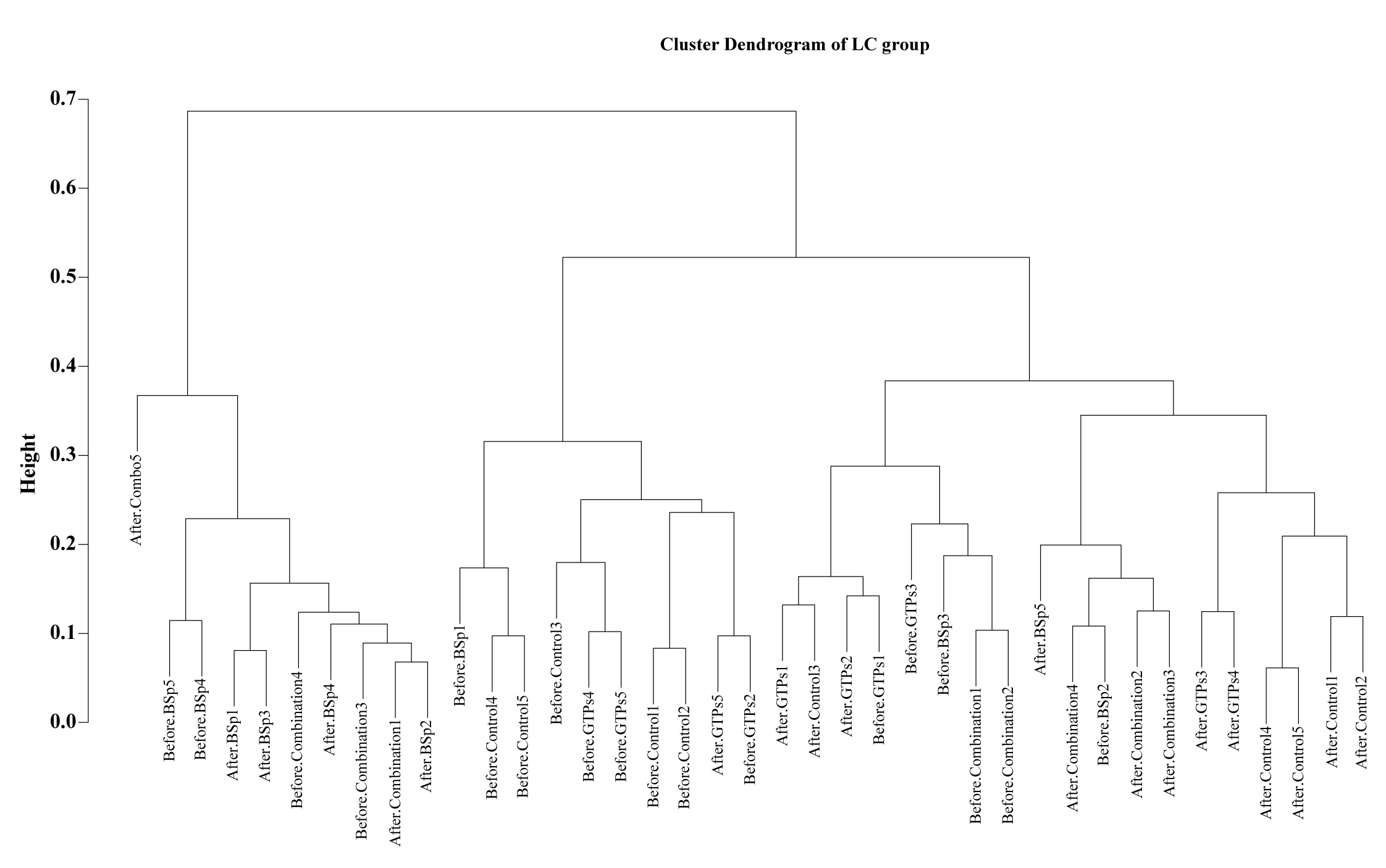

Supplement: S2 Fig — This hierarchical clustering dendrogram verifies that no outliers were present and we have therefore included every sample for microbiome analyses of the LC group. (TIF) [file pone.0234893.s002.tif]
